# Field metabolic rates of giant pandas reveal energetic adaptations

**DOI:** 10.1038/s41598-021-01872-5

**Published:** 2021-11-17

**Authors:** Wenlei Bi, Rong Hou, Jacob R. Owens, James R. Spotila, Marc Valitutto, Guan Yin, Frank V. Paladino, Fanqi Wu, Dunwu Qi, Zhihe Zhang

**Affiliations:** 1 Department of Biodiversity, Earth and Environmental Science, Drexel University, 3145 Chestnut St, Philadelphia, PA 19104 USA; 2 Sichuan Key Laboratory of Conservation Biology for Endangered Wildlife, Chengdu Research Base of Giant Panda Breeding, 1375 Panda Rd, Chengdu, 610081 Sichuan Province China; 3 Department of Conservation, Los Angeles Zoo, Los Angeles, CA 90027 USA; 4 Smithsonian Conservation Biology Institute, EcoHealth Alliance, 520 Eighth Avenue, Ste. 1200, New York, NY 10018 USA; 5 Department of Earth Sciences, Chengdu University of Technology, Chengdu, 610059 Sichuan Province China; 6 Department of Biology, Purdue University at Fort Wayne, 2101 E. Coliseum Blvd, Fort Wayne, IN 46805 USA; 7 Global Cause Foundation, 4031 University Drive #100, Fairfax, VA 22030 USA; 8 Sichuan Academy of Giant Panda, 1375 Panda RD, Chengdu, 610081 Sichuan Province China

**Keywords:** Physiology, Metabolism, Ecology, Conservation biology, Ecophysiology

## Abstract

Knowledge of energy expenditure informs conservation managers for long term plans for endangered species health and habitat suitability. We measured field metabolic rate (FMR) of free-roaming giant pandas in large enclosures in a nature reserve using the doubly labeled water method. Giant pandas in zoo like enclosures had a similar FMR (14,182 kJ/day) to giant pandas in larger field enclosures (13,280 kJ/day). In winter, giant pandas raised their metabolic rates when living at − 2.4 °C (36,108 kJ/day) indicating that they were below their thermal neutral zone. The lower critical temperature for thermoregulation was about 8.0 °C and the upper critical temperature was about 28 °C. Giant panda FMRs were somewhat lower than active metabolic rates of sloth bears, lower than FMRs of grizzly bears and polar bears and 69 and 81% of predicted values based on a regression of FMR versus body mass of mammals. That is probably due to their lower levels of activity since other bears actively forage for food over a larger home range and pandas often sit in a patch of bamboo and eat bamboo for hours at a time. The low metabolic rates of giant pandas in summer, their inability to acquire fat stores to hibernate in winter, and their ability to raise their metabolic rate to thermoregulate in winter are energetic adaptations related to eating a diet composed almost exclusively of bamboo. Differences in FMR of giant pandas between our study and previous studies (one similar and one lower) appear to be due to differences in activity of the giant pandas in those studies.

## Introduction

Giant pandas (*Ailuropoda melanoleuca*) are an icon of conservation and the flagship species for conservation in China. Because of their protection, numerous less well-known animals and plants are also protected in giant panda reserves^1^. It is of great concern that for such an important species so little is known about its basic physiology and ecology. To improve the conservation of giant pandas and better design their nature reserves we need more understanding of the limiting factors that control their distribution. Energy exchange is one of the most ubiquitous and influential relationships between an animal and its environment^2^. Factors such as radiation (sunlight, skylight, and radiant heat), air temperature, wind, and humidity affect the energy flow to and from an animal. In turn, the energy flow between an animal and the environment affects the metabolism and body temperature of the animal. A result of this dynamic is that animals typically live in the environment most suited to their physiological and energy requirements for energy balance^2^. Those individuals that can obtain and process energy with the greatest efficiency while balancing that task against other factors affecting their survival and reproduction will have the greatest genetic fitness^3^. Therefore, understanding the energy exchange of a giant panda will help us to better predict its success in nature.

Giant pandas are primarily herbivorous bears in the Family Ursidae. Other bears include the American black bear (*Ursus americanus*) an omnivore that eats primarily vegetation, but also insects, honey and meat when available; the Asiatic black bear (*Ursus thibetanus*) an omnivore with a similar diet; the sloth bear (*Ursus ursinus*) that eats termites; the sun bear (*Ursus malayanus*) that is an omnivore; the polar bear (*Ursus maritimus*) that is a carnivore, the brown or grizzly bear (*Ursus arctos*) that is an omnivore eating primarily vegetation but also hunting animals, catching fish (salmon) and eating carrion; and the Andean or spectacled bear (*Tremarctos ornatus*) that is primarily a herbivore. The giant panda is similar in size (males 100–160 kg and females 70–125 kg) to the Asiatic black bear (males 110–150 kg and females 65–90 kg), the sloth bear (males 80–145 kg and females 55–105 kg) and Andean bear (males 100–200 kg and females 35–82 kg). It is smaller than the American black bear (males 115–270 kg and females 92–170 kg), grizzly bear (males 180–400 kg and females 130–180 kg) and polar bear (males 350–700 kg and females 150–300 kg)^4^. Most bears, including giant pandas, are animals of the forest but before the arrival of Europeans, grizzly bears also inhabited the Great Plains of North America and polar bears are animals of the Arctic. All of these bears have a thick coat of insulation especially the grizzly and polar bears. It is possible that the habitat and unique diet of giant pandas may have led to unique specializations in energy expenditure.

Initially metabolic studies on bears focused on their reduced metabolism during hibernation^5–10^. Resting metabolic rates have been measured for sloth bears^11^, American black bears^10^, polar bears^7,12,13^, and grizzly bears^12^. In addition to the studies on giant pandas, field metabolic rates (FMR) have also been measured for polar bears^12,13^ and active metabolic rates have been reported for sloth bears^11^ and grizzly bears^12^. By comparing the metabolic rates of giant pandas in a natural environment with those measured in other bears we can assess whether giant pandas have a lower metabolic rate than expected based on allometric predictions.

Zhang et al.^14^ used a mechanistic model based on biophysical interactions to explore likely panda response to habitat alteration and climate change considering physiological, behavioral and ecological factors. Their analysis was limited by uncertainty about the metabolic rate of the giant panda. Fei et al.^15^. reported that the resting metabolic rate (RMR) of adult and sub-adult giant pandas measured with flow through respirometry in the laboratory was 0.150 ml/g/h, which was about 6 to 44% below the values for other mammals of similar size. The RMR of the giant pandas was 6.0 to 44.3% below those predicted by a regression line for carnivores/ungulates/pangolins (Fereuungulata) from Sieg et al.^16^. Nie et al.^17^ reported that giant pandas in captivity and the wild had FMR measured with doubly labeled water (DLW) as low as 1/3 the predicted value for mammals of their size and lower than the RMR of sleeping giant pandas in the laboratory reported by Fei et al.^15^ The FMR (also called daily energy expenditure DEE) is the average energy expended each day by an animal, including all of its physiological processes while it is active, feeding, breeding, and moving around in its environment^18–28^. However, Fei et al. reported that FMR was 0.284 ml/g/h, four times higher than for the captive animals in Nie et al. and 3.5 times higher than the rate for their wild animals. So, there is a conundrum that needs to be resolved. Are giant pandas low energy specialists or do they have a metabolism similar to that of other mammals of their size?

The Chengdu Research Base of Giant Panda Breeding is developing a release program to reintroduce captive bred giant pandas (*Ailuropoda melanoleuca)* into the wild. For this program we adapted an assisted soft release method, based largely on the soft release approach of Benjamin Kilham^29^ in New Hampshire, USA. This method links an investigator with a giant panda cub and provides an opportunity to collect more data on the physiology, behavior and ecology of pandas living in wild conditions via direct observations and measurements of undisturbed animals. Because of this relationship between the investigator and the giant panda we were able to inject DLW and withdraw blood samples on undisturbed animals that were fully awake. The release program pandas move from a standard zoo enclosure to large, naturally forested enclosures in a nature reserve where they have to forage for natural and provisioned bamboo, and finally into the wild habitat of the nature reserve. Our observations to date indicate that these pandas increase their activity to forage on natural growing bamboo. Also, giant pandas in large enclosures are more vigilant and regularly climb trees to avoid potential danger, increasing their activity compared to the highly habituated giant pandas in a zoo. Giant pandas released into the wild quickly adjust to the natural habitat and move about from one bamboo patch to another (personal observations).

It is now important to determine the FMR of giant pandas under these natural conditions in order to predict the number of giant pandas that can be released into various nature reserves. We have already measured the metabolic rates of giant pandas in captivity by flow through respirometry and DLW^15^. Those animals had metabolic rates measured by flow through respirometry similar to, but lower than similar sized mammals such as the tiger (*Panthera tigris*), lion (*Panthera leo*), cow (*Bos taurus*), and eland (*Taurotragus oryx*)^16^. Giant panda FMR in zoo enclosures were lower than some similarly sized mammals such as deer (*Odocoileus hemionus* and *Cervus elaphus*), and oryx (*Oryx leucoryx*), but higher than that of reindeer (*Rangifer tarandus*)^26^.

The objective of this study was to measure the metabolic rates of giant pandas in zoo enclosures and in large enclosures in nature reserves to compare the FMR of giant pandas under captive and more naturalistic conditions in order to answer the basic question: What is the FMR of a giant panda when living in naturalistic conditions?

## Methods

### Giant pandas

Giant pandas in this study were from the reintroduction program of the Chengdu Research Base of Giant Panda Breeding (Panda Base). We measured FMR of five pandas (2 pandas chosen for reintroduction and 3 control pandas) in enclosures at different study sites. The control pandas lived at the Panda Base and the other two pandas lived at Panda Valley and the Daxiangling Nature Reserve. In Panda Valley, a research facility in Dujiangyan, the release pandas ate the same species of harvested bamboo (stem, shoots, and leaves) as the control pandas in Panda Base. However, the release pandas also ate *Phyllostachys bissetii* bamboo that grew naturally inside a learning enclosure at Panda Valley. At Daxiangling Nature Reserve, the release giant pandas ate natural arrow bamboo (*Bashania faberi*) inside the learning enclosure. We also provided apples for the released giant pandas when we conducted blood sampling or body health checks. No other food was provided to the release giant pandas. The control captive pandas lived in a zoo enclosure and ate bamboo, bamboo shoots, apples, and “panda cake,” a biscuit made of a mixture of grains with vitamins at Panda Base. We measured FMR twice on each release panda at Panda Valley and Daxiangling Nature Reserve and once on each control panda at Panda Base zoo enclosure (Table 1).

**Table 1:** Water dose, experiment duration, dilution space and isotope turnover rates for giant pandas in the study of metabolic rates of giant pandas under natural conditions and at the Chengdu Research Base of Giant Panda Breeding in Chengdu, China.

Panda	Studbook number	Panda source	Dose (g)	Duration (day)	Nd (mol)	No (mol)	Nd/No	kd	ko	kd/ko
XingChen	1000	Project	8.134	3.76	2163.86	2024.43	1.07	0.0086	0.0099	0.8687
XingChen	1000	Project	9.025	3.81	2525.31	2554.93	0.99	0.0160	0.0179	0.8938
QianQian	881	Project	11.058	3.83	3949.87	3681.39	1.07	0.0092	0.0109	0.8440
QianQian	881	Project	11.596	3.81	3549.17	3475.72	1.02	0.0104	0.0129	0.8062
YuanRun	853	Captive	12.048	3.68	4141.63	4216.87	0.98	0.0061	0.0070	0.8714
QiYi	1008	Captive	9.940	3.77	3678.31	3442.24	1.07	0.0041	0.0050	0.8200
DaMei	1073	Captive	10.034	3.70	2824.28	2973.68	0.95	0.0055	0.0056	0.9821

Nd is isotope dilution space of D2 and No is isotope dilution space of O18. The kd is mean isotope turnover rate of D2 and ko is mean isotope turnover rate of O18.

### Chengdu panda base

Panda Base in Chengdu, Sichuan Province, was established in 1987 as a rescue and breeding facility for giant pandas. A nonprofit organization open to the general public, the main functions of the Panda Base were giant panda breeding, research, conservation, education, educational tourism, and giant panda reintroduction. Pandas lived in a 0.77 ha enclosure and ate bamboo provided by the husbandry staff. The elevation of the Panda Base was 524 m. The mean annual temperature was 16 °C and the annual rainfall was 1000 mm.

### Panda Valley

Panda Valley was located in Dujiangyan, a city west of Chengdu. It was a semi-wild facility of the Panda Base. We built a 3.8-hectare semi-wild learning enclosure for reintroduction pandas. Pandas in the experiment spent their time outside in the learning enclosure. The elevation of Panda Valley was 815 m. The mean annual temperature was 15.2 °C and the annual rainfall was 1200 mm. Pandas ate natural growing bamboo (*Phyllostachys bissetii*) and bamboo brought into the enclosure to supplement natural growing bamboo.

### Daxiangling nature reserve

Daxiangling Nature Reserve was in the western part of the Sichuan Basin. The highest peak was 3552 m and elevation ranged from 1500 to 3553 m^30^. The climate was humid, annual rainfall was typically 1300–2000 mm, and the mean annual temperature was 16 °C^31^. The reintroduction giant pandas lived in natural enclosures of 13.3 and 49.4 ha at an elevation of 2456–2495 m. Pandas ate naturally growing bamboo.

### Sample collection, analysis, and calculation

**Figure 1:**
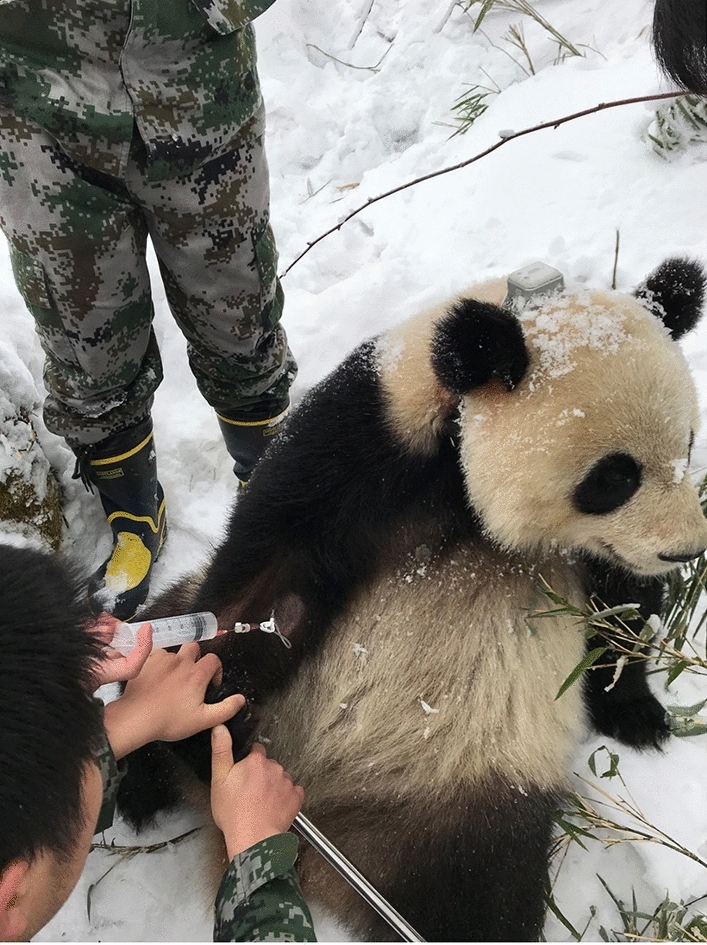
Injecting DLW into one of the female release giant pandas in winter at Daxiangling Nature Reserve, Sichuan Province, China. Pandas were awake and alert during injections and blood sampling and voluntarily offered their forelimb for sampling. Photo by Wenlei Bi.

To measure the FMR on wild pandas in the past, researchers used anesthetics to sedate the panda before the blood collections or injections^17^. We were concerned that sedation may lead to death^32^ as well as alter our results in the experiment^33,34^. In addition, while sedation of pandas under the supervision of trained veterinary staff is relatively safe, there is always a risk of morbidity or mortality, or disturbance of natural behaviors and physiological processes of the animal for hours or days afterward^35–37^. This risk is compounded in the field setting. Thus, to avoid sedation risks, we built a strong bond with our pandas at a young age. Even when our reintroduction pandas were released into the field, we could still touch and weigh them without using anesthetics or other restraint methods. We trained our experimental giant pandas to voluntarily grab a small pole with their front paw so that we could take a blood sample without anesthesia (Fig. 1). The DLW method based on the Fei et al.^15^ study was used to measure the metabolic rate. First, we took a 1 ml background blood sample. Then, we injected the panda with 8.1–12.1 g (Table 1) of DLW depending on the mass and time needed for the decay curve (Sigma-Aldrich deuterium oxide-18 (99% D 75% O^28^) mixed with physiological saline. Fei et al.^15^ determined that the equilibration time for giant panda was 5 h, so we took another 1 ml blood sample after 5 h. Finally, we took a 1 ml blood sample after 4 days. We sealed all blood samples in microcapillary glass tubes using an alcohol burner, placed them in a bigger PCV tube with cotton to protect them, and stored them in a freezer at – 40 °C. We sent the blood sample to the Laboratory of Isotope Geology at the Chengdu University of Technology for further analysis. We used the two-sample technique^38^ to calculate the CO_2_ production after 4 days. We used 0.9 as RQ to predict oxygen consumption because the giant pandas ate primarily carbohydrates (bamboo) but may have eaten some protein as well. The body mass versus FMR equation of Nagy et al.^39^ was used to predict FMR of a studied giant panda.

We used 24-gauge Cu-Co thermocouples (+/− 0.05 °C) to measure the air temperature in the study site. For the statistical analysis, we reported the results as mean ± SD.

The data were confounded by the small sample size and the fact that giant pandas varied in mass, lived at different altitudes and two were sampled twice. There were not enough data (only 7 data points) to do a 3 Way ANCOVA (mass, altitude, temperature) to test for the effect of those factors. We did do a regression analysis for both temperature and mass but neither was statistically significant because of the small sample size. We did not have an activity budget for the giant pandas at Daxiangling because they were usually hidden in bamboo thickets and we could not observe them 24 h a day. We compared the RMR and FMR of giant pandas to those of bears and other large mammals by constructing regression equations using Excel and comparing to the predicted FMR regression of Nagy (see “Discussion”).

### Animal use and care approval

This study was approved by the Institutional Animal Care and Use Committees of the Chengdu Research Base of Giant Panda Breeding (2019019) and Drexel University. All experiments were performed in accordance with the relevant guidelines of those committees. This study is reported in accordance with ARRIVE guidelines.

## Results

**Figure 2:**
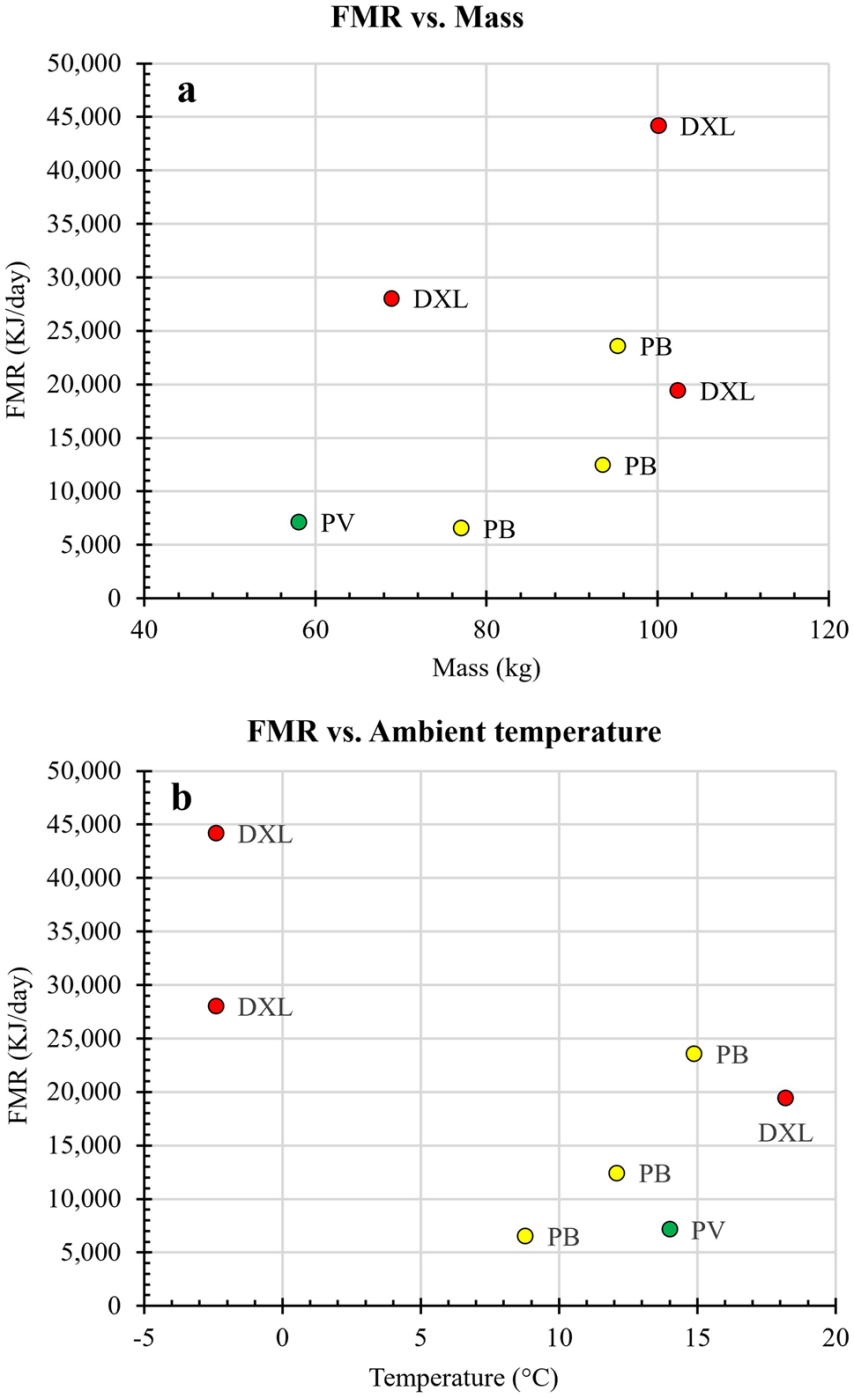
Relationship of field metabolic rates (FMR) of giant pandas measured with DLW to mass (**a**) and ambient temperature (**b**). We did the experiments at Research Base of Giant Panda Breeding in Chengdu, Sichuan Province, China (PB), at the Panda Valley facility of Panda Base in Dujiangyan, Sichuan Province, China (PV) and at the Daxiangling Nature Reserve, Sichuan Province, China (DXL). Elevated FMR of two pandas at DXL occurred at low ambient temperature as seen in 2b.

We measured the FMR using DLW in seven experiments on five individuals in three different seasons (Table 2). The highest FMR on a per unit basis was 0.89 ml O_2_/g/h for a panda at Daxiangling Nature Reserve in winter. The lowest FMR was 0.17 ml O_2_/g/h for a captive panda at Panda Base. The average FMR was 0.47 ml O_2_/g/h (SD = 0.26, range = 0.17–0.89 ml O_2_/g/h). The average FMR was 20,189 kJ/day (SD = 12,341, range = 6521–44,170 kJ/day). The highest FMR was from a release panda in winter (44,170 kJ/day) at the Daxiangling Nature Reserve. That panda also had the highest FMR on a per g basis. The lowest FMR was from the youngest captive panda (6521 kJ/day). That panda had the lowest FMR on a per g basis. Mass appeared to affect the FMR of giant pandas (Fig. 2a) but the regression of FMR on mass was not statistically significant due to the small sample size and the confounding effect of ambient temperature. Ambient temperature appeared to have a large effect on FMR (Fig. 2b) but a linear regression was not significant because of small sample size and the residuals were uninformative in determining the effect of ambient temperature on FMR. The FMR of captive pandas at Panda Base (524 m) at 8.8 to 14.9 °C (mean = 14,182 kJ/day) was similar to the FMR of release giant pandas in small and large natural enclosures in summer (815 m and 2475 m) at 14.0 and 18.2 °C (mean = 13,280 kJ/day). Giant pandas exposed to − 2.4 °C in a large enclosure at Daxiangling Nature Reserve in winter (21 January 2020) had the highest metabolic rates (0.82 and 0.89 ml O_2_/h/g) and highest FMR (28,045 and 44,170 kJ/day). The metabolic rate of one panda in winter was more than twice as high as its metabolic rate in summer and that of another panda was four times higher than its metabolic rate in autumn (Table 2, Fig. 2b).

**Table 2:** Field metabolic rates of 5 female giant pandas measured with DLW at different locations during different seasons.

Studbook number	Panda age	Season	Location	Mass (kg)	Ambient temperature (°C)	FMR CO_2_ ml/g/h	FMR O2 ml/g/h	FMR KJ/day	Predicted FMR KJ/day
1000	2.4	Autumn	Panda Valley	58.1	14.0	0.22	0.25	7143	15,133.9
1000	3.5	Winter	Daxiangling	68.9	− 2.4	0.74	0.82	28,045	17,151.4
881	5.0	Summer	Daxiangling	102.4	18.2	0.34	0.38	19,417	22,940.7
881	6.0	Winter	Daxiangling	100.1	− 2.4	0.80	0.89	44,170	22,561.4
853	7.3	Autumn	Panda base	95.4	14.9	0.45	0.50	23,592	21,778.9
1008	3.4	Winter	Panda base	93.6	12.1	0.24	0.27	12,435	21,476.5
1073	2.2	Winter	Panda base	77.1	8.8	0.15	0.17	6521	18,627.1

Pandas 881 and 853 are adults. The others are sub-adults. The FMR was calculated using an RQ of 0.9. The body mass vs. FMR equation of Nagy^39^ was used to predict FMR of a studied giant panda. The values for FMR (KJ/day) in this table differ from those in Bi (2020)^55^ (see Supplementary Information).

**Figure 3:**
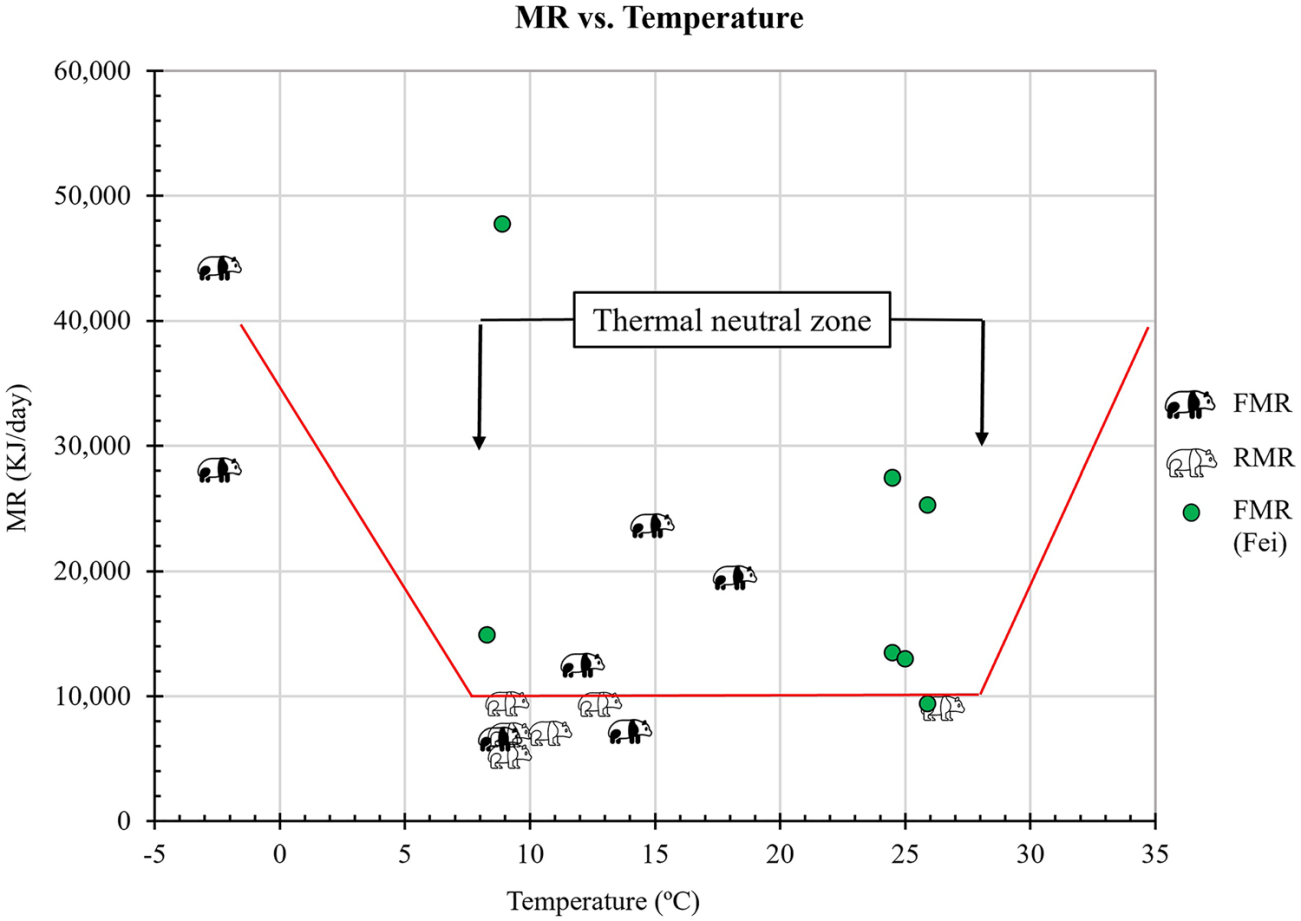
The thermal neutral zone of the giant panda as determined from field metabolic rates (FMR) measured in this study and resting metabolic rates (RMR) and FMR measured by Fei. et al.^15^. The lower critical temperature is 8 °C and the upper critical temperatrure is 28 °C. The horizontal red line represents the predicted thermal neutral zone of the giant panda (see arrows). Field metabolic rates only give an approximate indication of the thermal neutral zone because the giant pandas are active and not resting.

When we plotted the RMR reported by Fei et al.^15^ and the FMR measured in this study and by Fei et al.^15^ we were able to determine the thermal neutral zone of the giant panda. The thermal neutral zone is the range of temperatures in which a homeotherm maintains a constant metabolic rate^40^ by regulating its body temperature by insulation and vasomotor response alone. It appeared that the thermal neutral zone for the giant panda was between 8 °C and 28 °C (Fig. 3).

## Discussion

Measurement of FMR of giant pandas revealed energetic adaptations in summer and winter. Giant pandas at Panda Base, Panda Valley, and Daxiangling Nature Reserve at 8.8 to 18.2 °C had FMRs (mean = 0.314 ml O_2_/g/h) similar to those reported by Fei et al.^15^ for giant pandas at Panda Base at 8.3 to 25.9 °C (mean = 0.349 ml O_2_/g/h)). The effect of temperature was striking in our study. Two giant pandas at − 2.4 °C had FMR of 0.821 and 0.890 ml O_2_/g/h, several times higher than the same pandas at 14.0 °C and 18.2 °C in autumn and summer. Clearly, giant pandas were below their thermal neutral zone at freezing temperatures and had to raise their metabolic rate to thermoregulate under those conditions. Thus giant pandas have a similar thermoregulatory capacity for cold as a polar bear cub, Arctic ground squirrel (*Citellus parryii*)^41^,domestic rabbit^42^, and red panda (*Ailurus fulgens*)^43^ and less thermoregulatory capacity than a snowshoe hare (*Lepus americanus*), and Arctic fox (*Vulpes (Alopex) lagopus*)^40,41^, all much smaller mammals. Bears, relatives of giant pandas, go into torpor or hibernation when faced with low winter temperatures. Their body temperature drops to 31 °C to 36 °C and metabolism, fueled by body fat, decreases by 30–50%^43^. Giant pandas cannot store up enough body fat to hibernate and thus are active all winter eating bamboo^44^.

Our FMRs of giant pandas were similar to those measured by Fei et al.^15^. They found the average daily energy expenditure (FMR) of 7 captive giant pandas (body mass averaged 125 kg) was 21,592 kJ/day (SD = 13,323, range = 9401 kJ/day to 47,716 kJ/day), which is lower than those of some similar-sized mammals^26^ but higher than Nie et al.^17^ reported. Nie et al.^17^ found average FMR for 5 captive pandas (body mass average 91.1 kg) was 4579.92 ± 1887.05 kJ/day (± SEM) and for 3 wild pandas (body mass average 92.6 kg) was 6246.13 ± 2173.56 kJ/day. The FMR of our free-roaming giant pandas in large wild enclosures (13,280 kJ/day) was higher than the FMR reported for adult wild-caught pandas (6,246.13 kJ/day, SD = 2173.56) by Nie et al.^17^ despite the fact that they weighed less. Our results are generally consistent with those of Fei et al.^15^ and indicate that the metabolic rates of giant pandas under natural conditions are higher than those reported by Nie et al.^17^. It is not clear to us why the giant pandas studied by Nie et al. had much lower metabolic rates than those studied here and by Fei et al. One possibility is that there were large differences in activity between the pandas in our study and Fei’s study as compared to those in the Nei study. To determine if giant pandas have exceptionally low FMR it is necessary to compare the FMR of the giant panda to those of other bears.

**Figure 4:**
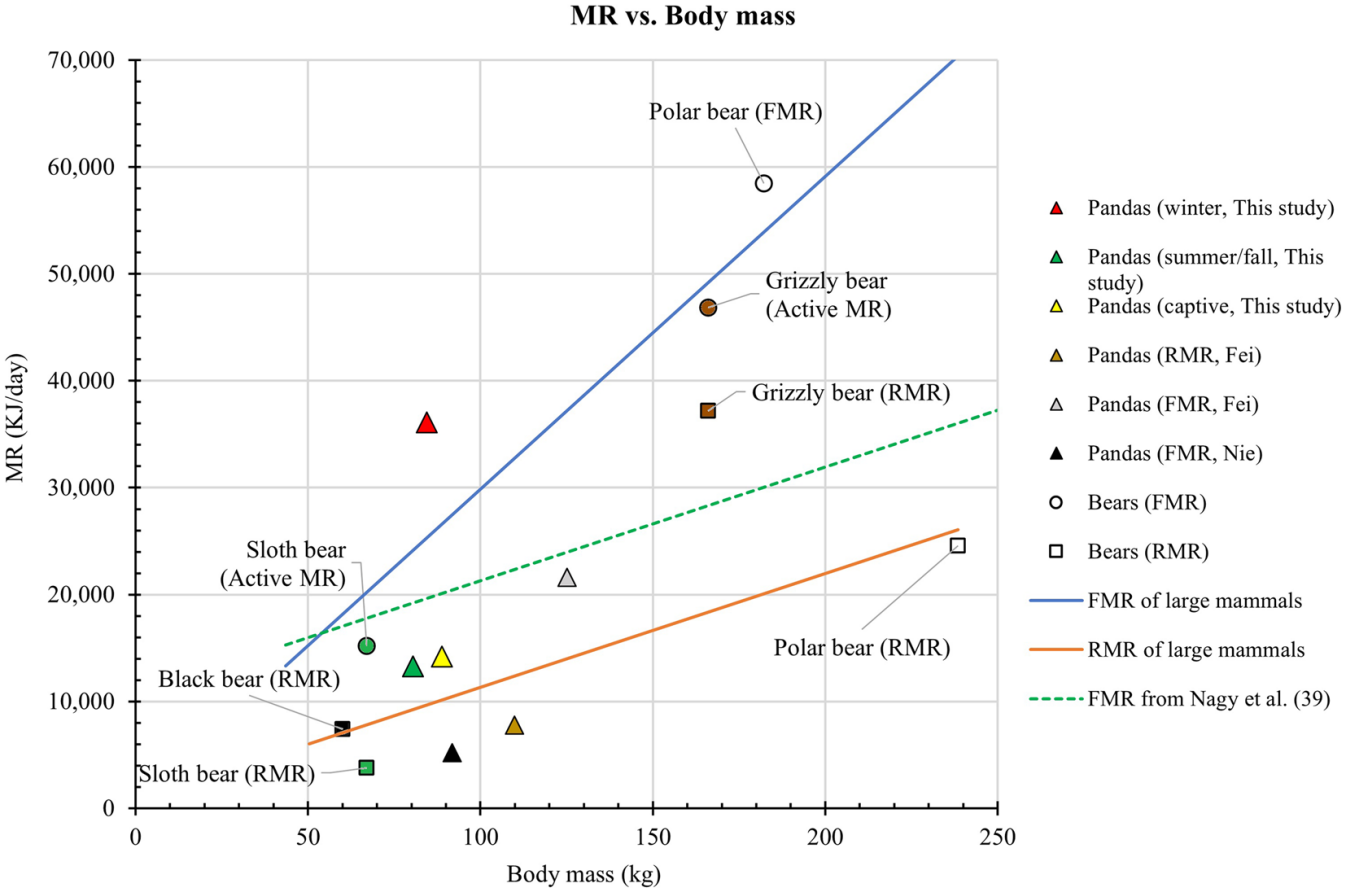
Relationship of metabolic rate and body mass in giant pandas and other bears. Metabolic rate includes field metabolic rate (FMR), resting metabolic rate (RMR), and active metabolic rate. The orange regression line for RMR of large mammals was calculated using the data in Table 3 and included the resting metabolic rates of 20 large mammals from Sieg et al.^16^ and average resting metabolic rates of grizzly bears and polar bears from Pagano et al.^12^, sloth bears from McNab^11^, and black bears from Toien^10^. It did not include the RMR of the giant panda. The regression equation is (RMR) = 106.41 (mass_kg_) + 671.77 (r^2^ = 0.524, *p* = 0.00), where y is RMR and x is body mass. The green dashed line is the regression line from Nagy et al.^39^ (FMR = 4.82 (mass_g_)^0.73^). The blue regression line for FMR of large mammals included the FMR of 10 large mammals and active metabolic rates and FMR of bears from the Table 4. It did not include the FMR of giant pandas. The regression equation is (FMR) = 292.66 (mass_kg_) + 585.35 (r^2^ = 0.99, *p* = 0.00) where y is FMR and x is body mass.

Resting metabolic rates of giant pandas measured with flow-through respirometry^15^ were higher than that of sloth bears^11^, similar to that of black bears^10^, but 63% of that predicted from a regression equation we calculated for large mammals (Fig. 4). Sloth bears had a RMR 48% of that predicted by that regression line while black bears had a RMR about as predicted. Polar bears had a RMR as predicted by that equation while grizzly bears had a much higher RMR than predicted^12,45^ (Table 3, Fig. 4). The FMRs of our pandas at 8.8 to 18 °C were lower than the active metabolic rate of sloth bears^11^ and 69% of the predicted FMR for mammals of that size calculated using the equation of Nagy et al.^39^. Fei’s giant pandas had a FMR 81% of that predicted by the Nagy et al. regression. (Table 4, Fig. 4). Both polar bears and grizzly bears had much higher FMR than those predicted by the Nagy et al. regression. The regression equation that we calculated for FMR based on data from large mammals produced a much steeper regression line (blue line in Fig. 4) than the regression line produced by the Nagy et al. equation (dashed green line in Fig. 4). That was because the largest mammal in the Nagy equation was about 100 kg and mammals we included in our regression (Table 4) included the large grizzly and polar bears (166 and 182 kg) and walruses (1310 kg). There are probably higher total energy costs for movement and activity in the large mammals than accounted for by the regression equation primarily based on smaller mammals although large mammals spend less energy per unit mass to move a given distance^45^. More data are needed for the FMR of large mammals to clarify these relationships.

**Table 3:** Resting metabolic rates of 20 large mammals compiled by Sieg et al.^16^, along with 4 bears and the giant panda.

Common name	Mass (kg)	RMR (KJ/day)
Grizzly bear (RMR)^12^	166	37,172
Polar bear (RMR)^12^	239	24,567
Sloth bear (RMR)^11^	67	3778
Black bear (RMR)^10^	60	7414
Giant panda (RMR)^15^	110	7775
Jaguar	50	5011
White tailed deer	51	5181
Ribbon seal	55	8893
Red deer	58	7351
Bighorn sheep	67	10,313
Homo sapiens	68	5999
American badger	76	10,214
Arabian oryx	84	8324
Caribou	85	13,171
Lion	98	7593
Water buck	100	11,958
Llama	115	8447
Sea lion	122	19,097
Eland	125	13,380
Tiger	138	10,746
Wildebeest	140	13,355
Harp seal	150	13,100
Bottlenose dolphin	166	24,849
Ass	178	13,037
Cow	193	15,126

**Table 4:** Field metabolic rates (FMR) and active metabolic rates of large mammals from this study*, Hudson et al.^26^ and other investigators. Summer data from our study includes pandas in summer and autumn from Table 2.

Common name	Mass (kg)	FMR (KJ/day)
Camelid	48	14,281
Fur seal	44	18,176
Grey kangaroo	61	10,730
Mule deer	53	34,698
Orangutan	114	53,868
Oryx	84	16,484
Red deer	108	25,249
Reindeer	75	8009
Sea lion	61	16,113
Walrus	1310	383,660
Giant panda (FMR)^15^	125	21,630
Giant panda (winter)*	85	36,108
Giant panda (summer)*	80	13,280
Giant panda (captive)*	89	14,182
Polar bear (FMR)^13^	182	58,470
Sloth bear (active MR)^11^	67	15,203
Grizzly bear (active MR)^12^	166	46,837
Giant panda (FMR)^17^	92	5205

The FMRs of giant pandas measured in our study were similar to the FMRs (ml O_2_/g/h) of polar bears measured with DLW although FMR expressed as KJ/day was much higher in polar bears since they were much larger than the giant pandas in our study^13^. In addition, polar bears were living at much colder temperatures in nature than our giant pandas. Pagano^46^ found that polar bears have higher energy expenditure in spring and that the spring ice conditions affect the prey availability of polar bears. Therefore, giant pandas and sloth bears had lower overall energy costs than polar bears and grizzly bears because they traveled much smaller distances than those bears and spent less energy on thermoregulation. The giant panda had a high FMR in winter at − 2.4 C because it was out of its thermal neutral zone. Optimal foraging theory predicts that animals will minimize their energy expenditure to obtain maximum food when foraging^47^. So, the release giant pandas in our study like sloth bears could be adapting their seasonal foraging by managing their energy expenditure as first suggested by Schaller et al.^44^. It would be interesting to measure the FMR of the American black bear since it has a low RMR but is an active forager and the Andean bear because it is a herbivore with greater home ranges than giant panda^4^. Meanwhile, we conclude that giant pandas do not have exceptionally low metabolic rates for bears, but like sloth bears they are low metabolic rate bears, with a somewhat reduced FMR due to their less energy intensive life style.

Our results on field metabolic rate (FMR) of giant pandas in captive enclosures and large enclosures in the Daxiangling Nature Reserve indicate that giant pandas must raise their metabolic rates to survive under cold conditions in winter. Giant pandas are not polar bears and have a much higher critical temperature. Previous statements that giant pandas are mountain animals acclimated to cold temperatures are perhaps overstated^48,49^. Schaller et al.^44^ noted that giant pandas in the Wolong Nature Reserve lived at higher altitudes because local people were farming the valleys and lower slopes. It is now clear that the habitat of the wild giant panda has been moved upward to higher elevations because of human disturbance^50–52^. Giant pandas live in only a small fraction of their historic range in areas that are remote and could not be easily farmed^53^. It is fortunate that giant pandas can survive in the mountains, but our results indicate that it is not their preferred thermal habitat.

Giant pandas have higher metabolic requirements at the montane elevations of the Daxiangling Nature Reserve (2500 m). That is not unexpected since other species also have higher metabolic requirements at high altitude. For example, deer mice (*Peromyscus maniculatus*) have a 75% greater mean FMR at high altitude (3800 m) than low altitude (1230 m)^54^. Fei et al.^15^ discussed the relationship between energy expenditure and natural bamboo resources and found that there is enough bamboo to fulfill the energy needs for wild giant pandas in an area such as Daxiangling. Our results support their analysis. Based on our FMR measurements and the fact that giant pandas eat about 20 kg of bamboo a day^15^, there is enough bamboo in 1 km^2^ of Daxiangling Nature Reserve to support 22 giant pandas. Since the density of giant pandas in the wild is much lower than that and their home range is between 3 and 6 km^2^, there must be other factors that restrict the number of giant pandas in a given area. In any case there is more than enough bamboo in nature reserves to support much larger populations of giant pandas than currently live there. Reintroduction efforts can proceed in releasing ever more giant pandas into reserves in order to repopulate natural areas with the certainty that there is a sufficient supply of bamboo to support those animals.

Preferably, nature reserves should be large and diverse in habitat and elevation so that more moderate climates are included, and giant pandas can descend the mountains during winter to find warmer microclimates. Conservation of giant pandas will require maintaining their genetic diversity in the wild in a natural biological community^53^. Moving towards that goal, reintroduction efforts should concentrate on releasing giant pandas into reserves such as Daxiangling that provide a large range of elevations (1500–3500 m) and thus microclimates for those pandas.

## Supplementary Information

Supplementary Information.

## Data Availability

All data are presented in this manuscript. For more information on the experiments contact JRS (Spotiljr@drexel.edu).

## References

[1] Li BV, Pimm SL (2016). China’s endemic vertebrates sheltering under the protective umbrella of the giant panda. Conserv. Biol..

[2] Porter WP, Gates DM (1969). Thermodynamic equilibria of animals with environment. Ecol. Monogr..

[3] Dunham AE, Grant BW, Overall KL (1989). Interfaces between biophysical and physiological ecology and the population ecology of terrestrial vertebrate ectotherms. Physiol. Zool..

[4] Nowak RM (1991). Walker’s Mammals of the World.

[5] Nelson RA, Wahner HW, Jones JD, Ellefson RD, Zollman PE (1973). Metabolism of bears before, during, and after winter sleep. Am. J. Physiol..

[6] Best RC (1982). Thermoregulation in resting and active polar bears. J. Comp. Physiol..

[7] Watts PD, Øritsland NA, Hurst RJ (1987). Standard metabolic rate of polar bears under simulated denning conditions. Physiol. Zool..

[8] Watts P, Cuyler C (1988). Metabolism of the black bear under simulated denning conditions. Acta Physiol. Scand..

[9] Watts PD, Jonkel C (1988). Energetic cost of winter dormancy in grizzly bear. J. Wildl. Manag..

[10] Tøien Ø (2011). Hibernation in black bears: Independence of metabolic suppression from body temperature. Science.

[11] McNab BK (1992). Rate of metabolism in the termite-eating sloth bear (*Ursus ursinus*). J. Mammal..

[12] Pagano AM (2018). Energetic costs of locomotion in bears: is plantigrade locomotion energetically economical?. J. Exp. Biol..

[13] Pagano AM, Williams TM (2019). Estimating the energy expenditure of free-ranging polar bears using tri-axial accelerometers: A validation with doubly labeled water. Ecol. Evol..

[14] Zhang Y, Mathewson PD, Zhang Q, Porter WP, Ran J (2018). An ecophysiological perspective on likely giant panda habitat responses to climate change. Glob. Change Biol..

[15] Fei Y (2016). Metabolic rates of giant pandas inform conservation strategies. Sci. Rep..

[16] Sieg AE (2009). Mammalian metabolic allometry: Do intraspecific variation, phylogeny, and regression models matter?. Am. Nat..

[17] Nie Y (2015). Exceptionally low daily energy expenditure in the bamboo-eating giant panda. Science.

[18] Acquarone M, Born EW, Speakman JR (2006). Field metabolic rates of walrus (*Odobenus rosmarus*) measured by the doubly labeled water method. Aquat. Mamm..

[19] Nagy K, Montgomery G (1980). Field metabolic rate, water flux, and food consumption in three-toed sloths (*Bradypus variegatus*). J. Mammal..

[20] Mautz W, Nagy K (1987). Ontogenetic changes in diet, field metabolic rate, and water flux in the herbivorous lizard *Dipsosaurus dorsalis*. Physiol. Zool..

[21] Anava A, Kam M, Shkolnik A, Degen A (2001). Effect of group size on field metabolic rate of Arabian babblers provisioning nestlings. Condor.

[22] Fyhn M (2001). Individual variation in field metabolic rate of kittiwakes (*Rissa tridactyla*) during the chick-rearing period. Physiol. Biochem. Zool..

[23] Møller AP (2008). Relative longevity and field metabolic rate in birds. J. Evol. Biol..

[24] Riek A (2008). Relationship between field metabolic rate and body weight in mammals: Effect of the study. J. Zool..

[25] Sparling CE, Thompson D, Fedak MA, Gallon SL, Speakman JR (2008). Estimating field metabolic rates of pinnipeds: Doubly labelled water gets the seal of approval. Funct. Ecol..

[26] Hudson LN, Isaac NJ, Reuman DC (2013). The relationship between body mass and field metabolic rate among individual birds and mammals. J. Anim. Ecol..

[27] Munn AJ (2016). Field metabolic rate, movement distance, and grazing pressures by western grey kangaroos (*Macropus fuliginosus melanops*) and Merino sheep (*Ovis aries*) in semi-arid Australia. Mamm. Biol..

[28] Drack, S. *et al*. Field metabolic rate and the cost of ranging of the red-tailed sportive lemur (*Lepilemur ruficaudatus*) in *New Directions in Lemur Studies* (eds. Rakotosamimanana, B., Rasamimanana H., Ganzhorn, J. U., & Goodman S. M.) 83–91 (1999).

[29] Kilham B, Gray E (2002). Among the Bears: Raising Orphan Cubs in the Wild.

[30] Xu W, Ouyang Z, Jiang Z, Zheng H, Liu J (2006). Assessment of giant panda habitat in the Daxiangling Mountain Range, Sichuan, China. Biodivers. Sci..

[31] Zhao C (2017). Relationship between human disturbance and endangered giant panda *Ailuropoda melanoleuca* habitat use in the Daxiangling Mountains. Oryx.

[32] Wysowski DK, Pollock ML (2006). Reports of death with use of propofol (Diprivan) for nonprocedural (long-term) sedation and literature review. J. Am. Soc. Anesthesiol..

[33] Mistraletti G, Donatelli F, Carli F (2005). Metabolic and endocrine effects of sleep deprivation. Essent. Psychopharmacol..

[34] Champagne CD, Houser DS, Costa DP, Crocker DE (2012). The effects of handling and anesthetic agents on the stress response and carbohydrate metabolism in Northern elephant seals. PLoS ONE.

[35] Fahlman, Å. *Anaesthesia of wild carnivores and primates*. Licentiate Thesis (Swedish University of Agricultural Sciences, Uppsala, Sweden, 2005).

[36] Arnemo JM (2006). Risk of capture-related mortality in large free-ranging mammals: experiences from Scandinavia. Wildl. Biol..

[37] West G, Heard D, Caulkett N (2014). Zoo Animal and Wildlife Immobilization and Anesthesia.

[38] Speakman JR (1997). Doubly Labelled Water: Theory and Practice.

[39] Nagy KA, Girard IA, Brown TK (1999). Energetics of free-ranging mammals, reptiles, and birds. Annu. Rev. Nutr..

[40] Prosser CL, Brown FA (1991). Comparative Animal Physiology, Environmental and Metabolic Animal Physiology.

[41] Scholander PF, Hock R, Walters V, Johnson F, Irving L (1950). Heat regulation in some arctic and tropical mammals and birds. Biol. Bull..

[42] Hart, J. S. Rodents in *Comparative Physiology of Thermoregulation, Volume II Mammals* (ed Whittow, G. C.) 1–149 (Academic Press, 1971).

[43] McNab BK (2002). The Physiological Ecology of Vertebrates: A View From Energetics.

[44] Schaller GB, Hu JC, Pan WS, Zhu J (1985). Giant Pandas of Wolong.

[45] Taylor C, Heglund N, Maloiy G (1982). Energetics and mechanics of terrestrial locomotion. I. Metabolic energy consumption as a function of speed and body size in birds and mammals. J. exp. Biol..

[46] Pagano, A. M. *Polar bear (Ursus maritimus) behavior and energetics: New metrics for examining the physiological impact of a changing Arctic environment.* Ph.D. Dissertation (University of California Santa Cruz, CA, 2018).

[47] Pyke GH, Pulliam HR, Charnov EL (1977). Optimal foraging: A selective review of theory and tests. Q. Rev. Biol..

[48] Hu JC (2001). Research on the Giant Panda.

[49] Liu G, Guan T, Dai Q, Li H, Gong M (2016). Impacts of temperature on giant panda habitat in the north Minshan Mountains. Ecol. Evol..

[50] Hull V (2014). Impact of livestock on giant pandas and their habitat. J. Nat. Conserv..

[51] Hull V (2016). Habitat use and selection by giant pandas. PLoS ONE.

[52] Li BV, Pimm SL, Li S, Zhao L, Luo C (2017). Free-ranging livestock threaten the long-term survival of giant pandas. Biol. Cons..

[53] Pan W (2014). A Chance for Lasting Survival: Ecology and Behavior of Wild Giant Pandas.

[54] Hayes JP (1989). Field and maximal metabolic rates of deer mice (*Peromyscus maniculatus*) at lowand high altitudes. Physiol. Zool..

[55] Bi, W. *Physiological ecology of soft-release giant pandas (Ailuropoda melanoleuca)*. PhD Dissertation. (Drexel University, Philadelphia, PA, 2020)

