# Supplementary material for: Field metabolic rates of giant pandas reveal energetic adaptations
Source: Sci Rep. 2021 Nov 17;11:22391. doi: 10.1038/s41598-021-01872-5 (PMC8599739; doi:10.1038/s41598-021-01872-5)
Supplement: Supplementary file 1 — Supplementary Information. [file 41598_2021_1872_MOESM1_ESM.pdf]

Supplementary Information

Field Metabolic Rates of Giant Pandas Reveal Energetic Adaptations

Wenlei Bi, Rong Hou, Jacob Owens, James R. Spotila, Marc Valitutto, Guan Yin,
Frank V. Paladino, Fanqi Wu, Dunwu Qi, Zhihe Zhang

In preparing this manuscript we discovered that there was an error in Table 3.2 of Bi, W. *Physiological ecology of soft-release giant pandas (Ailuropoda melanoleuca)*. PhD Dissertation. (Drexel University, Philadelphia, PA, 2020). The column labeled FMR (KJ/day) contained incorrect data. Upon further examination of the original data, we discovered that there had been a simple transcription error and the wrong data set was entered into that column. Figures 3.4 and 3.6 in that dissertation also contain those incorrect data. The data given in this article are correct as are the figures.
